# Spectrum and genotype–phenotype correlation of NR5A1 variants in 46,XY DSD: a systematic review and meta-analysis

**DOI:** 10.1530/EC-25-0783

**Published:** 2026-05-06

**Authors:** Renata T Dallago, Rafael Loch Batista, Sorahia Domenice, Vania dos Santos Nunes-Nogueira, Berenice Bilharinho Mendonca

**Affiliations:** ^1^ Developmental Endocrinology Unit, Endocrinology Division, Internal Medicine Department, Medical School, University of São Paulo (USP), São Paulo, Brazil; ^2^ Hormone and Molecular Genetics Laboratory (LIM/42), Hospital das Clínicas da Universidade de São Paulo, São Paulo, Brazil; ^3^ Universidade Estadual Paulista (UNESP), Faculdade de Medicina, Botucatu, Brazil

**Keywords:** disorders of sex development, 46,XY DSD, atypical genitalia, gonadal dysgenesis, steroidogenic factor 1, *NR5A1*

## Abstract

**Context:**

*NR5A1* encodes steroidogenic factor 1, a master regulator of adrenal and gonadal development. Pathogenic *NR5A1* variants are among the most common genetic findings in 46,XY differences of sex development (DSD), yet the broad phenotypic spectrum remains incompletely defined.

**Objective:**

This study aims to establish pooled estimates of key clinical outcomes and to clarify whether variant type predicts phenotype, pubertal course, or gender transition in *NR5A1*-related 46,XY DSD.

**Methodology:**

MEDLINE, Embase, and HGMD were systematically searched. Two reviewers independently screened, extracted, and appraised studies following the methodology of the Joanna Briggs Institute. Ninety-eight studies (312 individuals) were included; 35 series with ≥3 cases entered meta-analysis. Across studies, 85% presented atypical external genitalia and 15% female-like genitalia; sex of rearing was female in 54%. Spontaneous puberty occurred in 82% (95% CI: 45–96), and adrenal insufficiency in only 1.6%. All reported gender transitions were female to male (10%, 95% CI: 5–21). Missense variants represented 54%. Meta-regressions revealed no association between variant class and genital phenotype (odds ratio (OR): 1.25) or between phenotype and gender transition (OR: 0.46).

**Conclusion:**

*NR5A1*-related 46,XY DSD is a dynamic condition with high rates of spontaneous virilisation (82%) and minimal adrenal involvement (1.6%). The absence of clear genotype–phenotype correlations and the occurrence of female-to-male gender reassignment support a conservative, longitudinal, patient-centred model of care. Given the unpredictability of individual outcomes, the quantitative estimates from this review support a shift towards evidence-based, longitudinal care that prioritises patient autonomy by deferring irreversible decisions.

## Introduction

Differences of sex development (DSDs) are congenital conditions characterised by atypical development of chromosomal, gonadal, and anatomical sex. They are often detected at birth due to atypical genitalia or during adolescence owing to atypical pubertal development (1). Among affected individuals, up to 75% present a 46,XY karyotype (2).

The *NR5A1* gene, previously known as *Steroidogenic Factor 1* (SF1), is located on chromosome 9q33.3 and encodes the NR5A1/SF1 protein (OMIM 184757) (3). *NR5A1* plays a crucial role in the transcriptional regulation of genes involved in steroidogenesis and adrenal and gonadal development (4, 5). Pathogenic *NR5A1* variants account for approximately 10–20% of 46,XY DSD cases (3), making it one of the most frequent monogenic causes identified to date.

The first two mutations described in the *NR5A1* gene were identified in 46,XY patients with primary adrenal insufficiency, who were phenotypically female with complete gonadal dysgenesis (CGD) and persistent Müllerian ducts (6, 7). Since then, a remarkably broad phenotypic spectrum has been recognised, ranging from female to atypical genitalia, gonadal dysgenesis, and male infertility in 46,XY individuals to primary ovarian insufficiency in 46,XX women and, in some cases, extragonadal anomalies (5, 8, 9, 10, 11). Despite the well-established role of *NR5A1* in gonadal and adrenal development, the relationship between variant type and clinical expression remains unclear (12, 13).

Knowledge about gonadal function at puberty in *NR5A1*-related 46,XY DSD remains limited, in part because many reported individuals underwent gonadectomy in early childhood (14). In addition, long-term clinical follow-up into adolescence and adulthood has not been consistently reported in published cohorts. Moreover, spontaneous pubertal virilisation is frequently observed in 46,XY DSD individuals carrying *NR5A1* variants (14, 15). Testosterone production fluctuates throughout life in individuals with *NR5A1* variants, contrasting with the severely under-virilised external genitalia observed at birth and the spontaneous virilisation at puberty observed in several cases (3). Despite testosterone serum concentrations within the male reference range and spontaneous progression of puberty, male individuals frequently exhibit impaired testicular development. Therefore, testicular volume does not correlate well with preserved Leydig cell function at puberty. Notably, despite normal testosterone levels, LH and FSH levels are elevated in most individuals but further increase during the course of puberty (15). Moreover, normal testosterone responses to hCG stimulation indicate that NR5A1/SF-1 function may be less critical for steroidogenesis at puberty than during fetal life (16).

Taken together, these findings highlight persistent uncertainty regarding how the *NR5A1* variant type influences the external genital phenotype, pubertal development, and sex outcomes. Therefore, the objective of this study was to perform a systematic review and meta-analysis to characterise the phenotype spectrum of *NR5A1*-related 46,XY DSD and to evaluate genotype–phenotype correlations, with a focus on spontaneous puberty, sex of rearing, and gender transition.

## Methods

This systematic review was conducted according to the Joanna Briggs Institute methodology for systematic reviews of aetiology and risk (17) and is reported according to the Preferred Reporting Items for Systematic Reviews and Meta-Analyses (18). The review protocol was registered in the International Prospective Register of Systematic Reviews (PROSPERO) and is discussed below.

### Eligibility criteria

This systematic review included observational studies, considered prospective and retrospective cohort studies, cross-sectional studies, case series, and case report studies that met the following participants–exposure of interest–outcomes (‘PEO’) structure:

*Participants:* patients with allelic variants in the *NR5A1* gene and 46,XY DSD.

*Exposure of interest:* pathogenic, likely pathogenic or variant of uncertain significance (VUS) allelic variant in the *NR5A1* gene in 46,XY DSD patients.

*Outcomes:*

- Spontaneous puberty: spontaneous puberty was considered a single measurement of testosterone above 150 ng/dL and LH above 0.3 U/L (ICMA) or 0.6 U/L (IFMA) in subjects with signs of puberty. The testosterone cut-off of 150 ng/dL was selected because it is compatible with mid-pubertal development (approximately Tanner stage 3 in males), reflecting clear activation of the hypothalamic–pituitary–gonadal axis rather than early or borderline pubertal values (19, 20). Pubertal progression was assessed according to Tanner staging when explicitly reported. In cases where Tanner staging was not available, descriptive clinical documentation of virilisation (testicular enlargement, penile growth, pubic hair development, or voice deepening) was considered indicative of pubertal activation. Hypergonadotropic hypogonadism was considered in individuals with testosterone production less than 150 ng/dL and elevated gonadotropins.
- Change in gender assignment: spontaneous change in gender assignment was considered when it occurred after individuals expressed desire.
- External genitalia: for the external genitalia classification, the following criteria were used:
  - Female-like: typical female external genitalia.
  - Atypical: presenting with any degree of virilisation, such as clitoromegaly, hypospadias, partial labial fusion, or micropenis.
- Variant classification: the types of allelic variants were divided into the following:
  - Missense variants.
  - Nonmissense variants (nonsense, splicing, small indels, and copy number variations – CNVs).

This binary classification was adopted to harmonise heterogeneous variant annotations across studies and to avoid sparse-data bias that would arise from multiple small subgroups in a rare condition. In addition, a descriptive analysis was performed in which variants were further subdivided into missense, in-frame indels, and probable loss-of-function variants and analysed according to their location within the functional domains of the NR5A1 protein (DNA-binding domain (DBD), hinge, and ligand-binding domain (LBD) to explore potential genotype–phenotype relationships.

The meta-analysis was restricted to 46,XY DSDs due to the larger number of available cohorts and to maintain comparable outcome metrics, as the inclusion of 46,XX cases would increase heterogeneity. Notably, *NR5A1* variants in 46,XX individuals are associated with a distinct and variable phenotype, primarily characterised by primary ovarian insufficiency (POI) with preserved female genital development. The clinical spectrum ranges from primary amenorrhoea with subsequent infertility to secondary amenorrhoea with preserved fertility followed by premature menopause (21).

### Identification of studies

#### Electronic databases

We systematically searched the following electronic databases: Embase (Elsevier), MEDLINE (via PubMed), and the Human Gene Mutation Database (HGMD). The databases were searched on June 18, 2024, without restrictions on language or publication year. The search strategy combined controlled vocabulary and free-text terms, including *disorders of sex development*, *disorder of sex development 46,XY*, *gonadal dysgenesis 46,XY*, *steroidogenic factor 1*, *SF-1 protein, human*, and *NR5A1 gene, human* (see Supplementary Table 1 for full search syntax (see section on Supplementary materials given at the end of the article)).

All retrieved records were exported into Mendeley Desktop (version 1.19.8) for reference management and automatic duplicate removal. Titles and abstracts were screened via the web-based application Rayyan QCRI (20) by two independent reviewers (22).

#### Study selection, data extraction, and risk-of-bias evaluation

Two reviewers (RTD and RLB) independently selected the titles and abstracts identified during the literature search. The full texts of potentially eligible studies were assessed for inclusion according to predefined PEO criteria (population, exposure, and outcome). Both reviewers used a standardised extraction form to assess all data from each study (type of variant, genitalia classification, assigned sex, change in gender assignment, social sex, and pubertal outcome). The risk of bias for each study was assessed via the Joanna Briggs Institute (JBI) checklist for case series (17). The overall methodological quality was good for most domains, except for the completeness of outcome reporting, as the lack of long-term follow-up was a recurrent limitation across studies. The detailed assessment is shown in Supplementary Table 2.

Any disagreements between reviewers regarding study selection, data extraction, or risk-of-bias assessment were resolved through discussion and consensus with the project coordinator (BBM).

#### Synthesis and analysis of data

All the statistical analyses were performed via Stata Statistical Software, version 18 (StataCorp LLC, USA), to plot similar outcomes in at least two studies included in the meta-analyses. The overall proportions for dichotomous outcomes were estimated via proportional meta-analysis. We used the updated command ‘metaprop_one’ and fit the logistic normal random effects model to the data (23). The number of events was used as the numerator, and the number of patients with allelic variants in the *NR5A1* gene and 46,XY DSD were used as the denominator. To avoid underestimating spontaneous puberty, for these outcomes, we used the number of patients in which pubertal outcomes were evaluated as the denominator. Continuous data were calculated as the means and standard deviations, and the pre- and post-exposure mean differences were calculated with respective 95% confidence intervals (CIs).

The odds ratio (OR) was used as a measure of association for dichotomous outcomes, enabling comparisons between exposed and unexposed groups regarding the outcomes of the event of interest. For each included study, the OR was either extracted directly or calculated from the absolute frequencies reported.

To assess inconsistencies across studies, forest plots were visually inspected, and statistical heterogeneity was quantified via the Higgins *I*^2^ statistic and the chi-square (*Q*) test. Heterogeneity was considered when *P* < 0.10 (*Q* test) and the *I*^2^ value >50%. When heterogeneity exceeded *I*^2^ > 30% and at least five studies were available, a prediction interval was calculated for the random effects meta-analysis. To explore potential sources of heterogeneity, meta-regression analyses were performed using study-level aggregated data. These analyses assess variation across studies rather than testing associations at the individual participant level. Meta-regressions were conducted only for outcomes with significant unexplained heterogeneity and a sufficient number of studies (≥10), specifically atypical genitalia, typical female genitalia, and spontaneous puberty. Two covariates were examined: the proportion of nonmissense variants and the proportion of missense variants.

Only studies including three or more patients were included in the meta-analysis to reduce imprecision associated with very small series (≤2 patients).

#### Quality of evidence

The quality of evidence regarding the association between *NR5A1* variants and phenotypic outcomes in 46,XY DSD individuals was assessed via the Grading of Recommendations, Assessment, Development, and Evaluation (GRADE) approach for observational studies (24).

## Results

### Study selection

**Figure 1:**
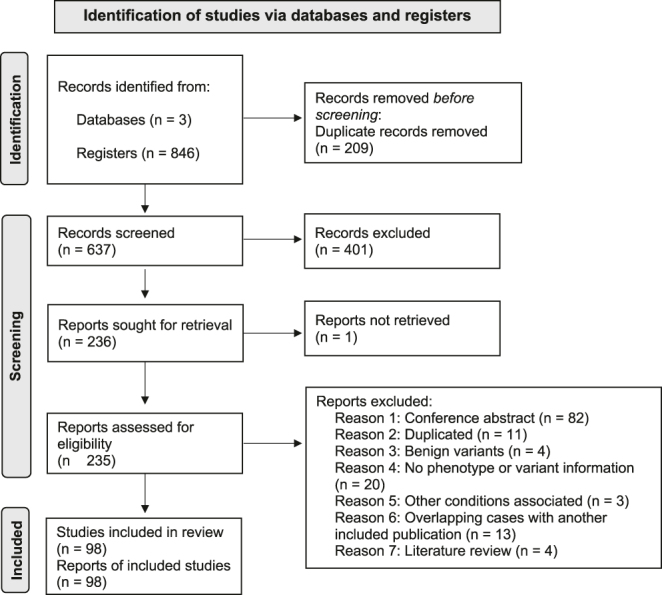
PRISMA flow chart outlining the process of study identification and selection.

The search strategies yielded many records. After removing duplicates, 637 unique studies remained for screening. Of these, 235 were considered potentially eligible and underwent full-text assessment. Following a detailed evaluation, 98 studies met all eligibility criteria and were included in the systematic review (3, 5, 6, 7, 8, 13, 14, 15, 16, 21, 25, 26, 27, 28, 29, 30, 31, 32, 33, 34, 35, 36, 37, 38, 39, 40, 41, 42, 43, 44, 45, 46, 47, 48, 49, 50, 51, 52, 53, 54, 55, 56, 57, 58, 59, 60, 61, 62, 63, 64, 65, 66, 67, 68, 69, 70, 71, 72, 73, 74, 75, 76, 77, 78, 79, 80, 81, 82, 83, 84, 85, 86, 87, 88, 89, 90, 91, 92, 93, 94, 95, 96, 97, 98, 99, 100, 101, 102, 103, 104, 105, 106, 107, 108, 109, 110, 111). A total of 137 studies were further excluded for the following reasons: conference abstracts (*n* = 82), duplicated studies (*n* = 11), benign variants (*n* = 4), lack of phenotype or *NR5A1* variant information (*n* = 20), associations with other conditions such as syndromes (*n* = 3), overlapping cases with another included publication (*n* = 13), and literature reviews (*n* = 4) (Fig. 1; Supplementary Material).

### Characteristics of the included studies

A total of 98 studies published between 1999 and 2024 were included in our systematic review, comprising 312 individuals with 46,XY DSD and confirmed allelic variants in the *NR5A1* gene. Of these, 35 were case series reporting data from three or more patients and were eligible for meta-analyses. The key clinical and demographic features of the study populations are summarised in Table 1.

**Table 1:** Main characteristics of studies included in the meta-analysis.

Authors (year)	*n* total	*n* hetero	*n* homo	*n* missense	*n* others	*n* fem	*n* masc	*n* gender change	*n* atypical	*n* female like	*n* desfPub	*n* pubEsp	*n* hipogon	*n* gonadect	*n* adrenal dysfunct
Achermann *et al.* (1999)	1	1	0	1	0	1	0	0	0	1	1	0	0	1	1*
Achermann *et al.* (2002)	1	0	1	1	0	1	0		0	1					1*
Adachi *et al.* (2018)	1	1	0	1	0	1	0	0	0	1	1	1	0	0	0
Alhamoudi *et al.* (2022)	1	1	0	1	0	1	0	0	1	0	1	1	0	0	0
Allali *et al.* (2011)	4	4	0	3	1	1	3	0	4	0					0
Alvaro *et al.* (2020)	1	1	0	1	0	0	1	0	1	0	1	1	0	0	0
Baetens *et al.* (2014)	3	3	0	1	2	0	3	0	3	0					0
Barbaro *et al.* (2011)	1	1	0	0	1	1	0	0	0	1	1	1	0	0	0
Bashamboo *et al.* (2016)	1	1	0	1	0	1	0	0	1	0					0
Bertelloni *et al.* (2014)	2	2	0	1	1	2	0	0	1	1	1	0	0	1	0
Brandt *et al.* (2013)	1	1	0	0	1	1	0	0	0	1					0
Buonocore *et al.* (2019)	5	5	0	4	1	5	0		5	0					0
Camats *et al.* (2012)	8	8	0	3	5	6	2	2	6	2	2	1	0	1	0
Chan *et al.* (2015)	1	1	0	1	0	1	0	0	0	1					0
Chauhan *et al.* (2017)	2	2	0	0	2	1	1	0	1	1					0
Cheng *et al.* (2021)	1	1	0	1	0	0	1	0	1	0					0
Ciaccio *et al.* (2012)	3	3	0	3	0	1	2	1	3	0	2	2	0	0	0
Colson *et al.* (2017)	2	2	0	0	1	1	0	0	1	1	2	2	0	0	0
Cools *et al.* (2012)	1	1	0	0	1	1	0	1	1	0	1	1	0	0	0
Cools *et al.* (2024)	13	9	2	6	7	6	7	0	10	3	1	1	0	0	0
Correa *et al.* (2004)	1	1	0	0	1	1	0	0	1	0	1	0	1	0	0
Coutant *et al.* (2007)	2	2	0	0	2	2	0	0	2	0	2	0	0	2	0
Damji *et al.* (2023)	1	1	0	1	0	1	0	0	1	0					0
Del gobbo *et al.* (2024)	4			0	4				4	0					0
Domenice *et al.* (2016)	10	10	0	6	4	9	1	1	9	1	8	5	0	3	0
Eggers *et al.* (2015)	3	3	0	0	3	1	2	0	3	0	1	1	0	0	0
Fabbri *et al.* (2016)	3	3	0	0	3	2	1	1	3	0	3	2	0	1	0
Fabbri-scallet *et al.* (2022)	3	3	0	0	3	0	3	0	3	0	2	2	0	0	0
Fabbri-scallet *et al.* (2018)	7	7	0	5	2	3	4	3	7	0	5	5	0	0	0
Fabbri-scallet *et al.* (2020)	9	5	2	7	2	3	6	0	8	1					0
Faienza *et al.* (2020)	4	4	0	4	0	2	2	0	2	2	4	4	0	0	0
Gau *et al.* (2023)	1	1	0	1	0			0	1	0					0
Gunes *et al.* (2023)	1	1	0	0	1	1	0	0	1	0	1	1	0	0	0
Harrison *et al.* (2013)	1			0	1	1	0	1	1	0					0
Hasegawa *et al.* (2004)	1	1	0	1	0	1	0	0	1	0	1	1	0	0	0
Hattori *et al.* (2017)	2	2	0	0	2	0	2	0	2	0					1^†^
Hu *et al.* (2012)	2	2	0	2	0	0	2		2	0					0
Hussain *et al.* (2016)	3	1	2	0	3	1	2	0	3	0					0
Kim *et al.* (2017)	1	1	0	1	0	1	0	0	0	1	1	0	0	1	0
Klrkgöz *et al.* (2024)	1	0	1	1	0	1	0		1	0					0
Kohler *et al.* (2008)	5	4	0	2	3	0	5	0	4	1	5	0	1	4	0
Kohler *et al.* (2009)	3	3	0	0	3	1	2	1	3	0					0
Laan *et al.* (2021)	2	2	0	0	2	0	2	0	2	0	2	2	0	0	0
Li *et al.* (2011)	2	2	0	0	2	2	0	0	0	2	2	0	2	0	0
Liao *et al.* (2011)	1	1	0	0	1	1	0	0	0	1					0
Lin lin *et al.* (2007)	4	4	0	4	0	3	1	0	3	1	4	1	0	3	0
Liu *et al.* (2019)	1	1	0	0	1	1	0	0	0	1					0
Lourenço *et al.* (2009)	3	3	0	1	2	2	1	0	1	2	2	1	1	0	0
Malikova *et al.* (2014)	5	5	0	3	2	1	4	1	5	0					1*
Mallet *et al.* (2004)	1	1	0	0	1	0	1	1	1	0	1	0	0	1	0
Mazen *et al.* (2016)	1	1	0	0	1	1	0	0	0	1	1	0	1	0	0
Monig *et al.* (2022)	7	7	0	2	5	6	1	3	5	2	7	7	0	0	0
Na *et al.* (2020)	10	9	1	5	5	5	5		8	2					0
Nagy *et al.* (2019)	1	1	0	0	1	1	0	0	1	0	1	0	0	1	0
Nishina-Uchida *et al.* (2013)	6			1	5	5	1		4	2	6	0	0	6	0
Ochoa *et al.* (2021)	3	3	0	3	0	0	3	0	3	0					0
Orekhova *et al.* (2018)	1	1	0	1	0	1	0	0	0	1					1^†^
Pan *et al.* (2020)	1	1	0	0	1	0	1	0	1	0					0
Paris *et al.* (2011)	1	1	0	1	0	0	1	0	1	0					0
Pedace *et al.* (2014)	1	1	0	0	1	0	1	0	1	0	1	1	0	0	0
Peycelon *et al.* (2017)	1	1	0	0	1	0	1	0	1	0	1	1	0	0	0
Philibert *et al.* (2007)	1	1	0	1	0	0	1	0	1	0	1	0	1	0	0
Philibert *et al.* (2011)	19	5	0	15	4				9	10	1	1	0	0	0
Ramos *et al.* (2024)	1	1	0	1	0	1	0	1	1	0					0
Reuter *et al.* (2007)	1	1	0	1	0	1	0	0	1	0	1	0	0	1	0
Robevska *et al.* (2018)	14	14	0	8	6	7	7		12	2	1	1	0	0	0
Rocca *et al.* (2018)	6	5	1	6	0	5	1	1	6	0	4	0	0	4	0
Shi *et al.* (2023)	1	1	0	1	0	1	0	0	1	0					0
Siklar *et al.* (2014)	1	1	0	0	1	1	0	0	0	1	1	1	0	0	0
Silfhout *et al.* (2009)	1			0	1	1	0	0	1	0					0
Soardi *et al.* (2010)	1	0	1	1	0	1	0	0	0	1					0
Song *et al.* (2018)	30	30	0	20	10	19	11	16	28	2	8	8	0	0	0
Srivastava *et al.* (2023)	2	2	0	0	2	2	0	1	2	0	1	1	0	0	0
Srivastava *et al.* (2022)	1	1	0	0	1	0	1	1	1	0	1	1	0	0	0
Sudhakar *et al.* (2019)	3	1	0	3	0	2	1		3	0	1	1	0	0	0
Suwanai *et al.* (2013)	2	2	0	0	2	1	1	0	2	0	1	0	0	1	0
Swartz *et al.* (2017)	2	2	0	0	2	0	2	0	2	0					0
Tajima *et al.* (2009)	1	1	0	1	0	1	0	0	1	0					0
Tantawy *et al.* (2014)	3	3	0	2	1	0	3	0	3	0	1	0	1	0	0
Tantawy *et al.* (2012)	2	2	0	2	0	1	1	0	1	1	2	2	0	0	0
Teoli *et al.* (2023)	1	1	0	0	1	0	1	0	1	0	1	1	0	0	0
Tuhan *et al.* (2017)	1	1	0	1	0	0	1	0	1	0	1	1	0	0	0
Wacharasindhu *et al.* (2023)	1	1	0	0	1	0	1	0	1	0					0
Wang *et al.* (2013)	1	1	0	1	0	1	0	1	1	0	1	1	0	0	0
Wang *et al.* (2020)	1	1	0	1	0	1	0	0	0	1	1	0	1	0	0
Warman *et al.* (2011)	6	6	0	0	6	2	4	0	6	0	4	2	1	1	0
Werner *et al.* (2015)	1	1	0	1	0	1	0	0	0	1	1	1	0	0	0
Werner *et al.* (2017)	4	4	0	1	3	3	1	1	3	1	3	2	1	0	0
Woo *et al.* (2015)	5	5	0	3	2	4	1	0	3	2					0
Wu *et al.* (2013)	1	1	0	1	0	0	1	0	1	0					0
Xia *et al.* (2021)	2	2	0	1	1	2	0	0	2	0	2	2	0	0	0
Yagi *et al.* (2015)	2	2	0	2	0	0	2	0	2	0	2	2	0	0	0
Yu *et al.* (2018)	13	13	0	8	5	11	2		5	8	3	3	0	0	0
Zangen *et al.* (2014)	1	0	1	1	0	1	0	0	0	1	1	0	1	0	0
Zhang *et al.* (2022)	1	1	0	1	0	1	0	1	1	0					0
Zhang *et al.* (2023)	1	1	0	1	0	1	0	0	0	1	1	0	1	0	0
Zheng *et al.* (2023)	4	4	0	3	1	3	1	0	1	3					0
Zidoune *et al.* (2022)	1	1	0	1	0	0	1	0	1	0					0

hetero, heterozygous; homo, homozygous; others, nonsense, splicing, small indels, and copy number variations; *n* fem, female sex assigned; *n* masc, male sex assigned; desfPub, pubertal outcome; *n* pubEsp, spontaneous puberty; *n* hipogon, hypergonadotropic hypogonadism; *n* gonadect, prepubertal gonadectomy; *n* adrenal dysfunct, adrenal dysfunction.

* Adrenal insufficiency.

^†^ Adrenal dysfunction = elevated ACTH with normal cortisol production; transient adrenal insufficiency in the first months of life.

### Meta-analyses

To increase the statistical precision and minimise the impact of small sample sizes, the meta-analyses were restricted to 35 studies that included three or more patients (3, 4, 13, 15, 16, 21, 30, 32, 33, 36, 38, 42, 43, 46, 48, 50, 51, 52, 53, 54, 55, 61, 68, 70, 71, 79, 80, 85, 92, 98, 100, 104, 108, 110, 112). The main features of the studies included in the meta-analysis are listed in Table 2.

**Table 2:** Main clinical features in 46,XY DSD patients with *NR5A1* variants.

Features	*n* (studies)	Overall frequency (%)	95% CI	Subset ≥ 3 patients* (%)	95% CI
Missense variants	312 (98)	53	41–64	54	42–66%
Atypical genitalia	312 (98)	81	73–87	85	75–91%
Female-like genitalia	312 (98)	19	13–27	15	09–25%
Female sex-assignment	312 (98)	_	_	54	42–66%
Spontaneous puberty	121 (59)	77	46–93	82	45–96%
Hypergonadotropic hypogonadism	121 (59)	4	0–4	7	03–15%
Prepubertal gonadectomy	121 (59)	1	0–78	6	01–44%
Gender transition (F → M only)	234 (87)	9	5–17	10	5–21%
Adrenal dysfunction	312 (98)	1.6 (5 patients)	_	_	_

* Group used for meta-analysis.

### Genotype

The overall proportion of missense variants was 54% (235 patients from 35 studies; 95% CI: 42–66%; *χ*^2^ = 8.9; *P* = 0.001). The remaining 46% comprised nonsense, splicing, small indel, and copy number variations (95% CI: 34–58%; *χ*^2^ = 8.9; *P* = 0.001).

**Figure 2:**
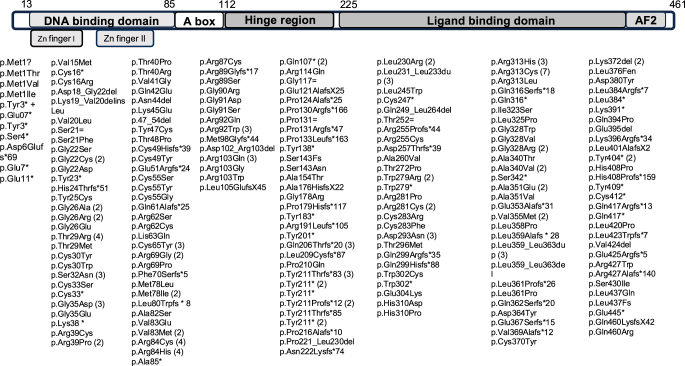
Schematic distribution of *NR5A1* variants identified in 46,XY DSD individuals. Schematic representation of the NR5A1/SF1 protein structure and distribution of reported variants across functional domains. The protein is shown from the N- to the C-terminus, highlighting the DNA-binding domain (including the zinc finger), A-box, hinge region, ligand-binding domain, and activation function-2 (AF-2).

In a descriptive analysis, heterozygous variants were identified in 302 cases, whereas homozygous variants were reported in ten individuals. Analysis of the variant distribution across the *NR5A1* gene revealed variant clustering at positions 84 (DBD), 211 (hinge region), and 313 (LBD) (Fig. 2).

### External genitalia phenotype and sex assignment

The overall proportion of female sex assignments was 54% (186 patients from 33 studies; 95% CI: 42–66%; *χ*^2^ = 9.2; *P* = 0.001), whereas 46% were male (95% CI: 34–58%; *χ*^2^ = 8.9; *P* = 0.001). Atypical external genitalia were observed in 85% of the cases (235 patients from 35 studies; 95% CI: 75–91%; *χ*^2^ = 14.3; *P* < 0.001), whereas typical female genitalia were reported in 15% (95% CI: 09–25%; *χ*^2^ = 14.3; *P* < 0.001), all of which were assigned as female. The overall proportion of change in gender assignment was 10% (161 patients from 28 studies; 95% CI: 5–21%; *χ*^2^ = 14.2; *P* < 0.001) (Table 2) (Supplementary Material). The descriptive data indicated that 24 individuals experienced sex transition, all of whom had atypical external genitalia at birth. Importantly, all the transitions occurred from female to male. Among the 24 individuals who underwent female-to-male sex transition, one had undergone prepubertal gonadectomy, while the remaining individuals retained their gonads until or during pubertal development.

In a descriptive analysis, variants were distributed across the three major functional domains of the NR5A1 protein, including the DBD, hinge region, and LBD. Female external genitalia were observed in 24.8% of the variants located in the DBD (28/113), 20.8% in the hinge region (11/53), and 20.7% in the LBD (24/116), whereas atypical genitalia were present in 75.2, 79.2, and 79.3% of the cases, respectively. The distribution of external genital phenotype across protein domains (DBD, hinge, and LBD) did not differ significantly (*χ*^2^ = 1.02; *P* = 0.60), indicating no association between variant location and phenotypic presentation. When variants were stratified according to predicted molecular consequences, female external genitalia and atypical genitalia were observed across all variant types. Female genitalia were present in 22.4% of missense variants (38/170), 23.1% of in-frame indels (3/13), and 20.1% of putative loss-of-function variants (26/129), with atypical genitalia accounting for the remaining cases. No significant differences were observed in the distribution of external genital phenotype across variant types (missense, in-frame indel, and loss-of-function) (*χ*^2^ = 0.27; *P* = 0.87), suggesting no association between variant class and phenotypic presentation.

### Pubertal outcome

The overall proportion of spontaneous puberty was 82% (78 patients from 23 studies; 95% CI: 45–96%; *χ*^2^ = 24.0; *P* < 0.001). Hypergonadotropic hypogonadism was observed in 7% (95% CI: 3–15%), and prepubertal gonadectomy was observed in 6% (95% CI: 1–44%; *χ*^2^ = 24.0; *P* < 0.001) (Table 2) (Supplementary Material).

In a descriptive analysis, when variants were subdivided according to the functional domains of the NR5A1 protein, individuals who had undergone prepubertal gonadectomy were excluded from the analysis. Overall, spontaneous puberty occurred in 81.4% of the individuals. Stratified by protein domain, spontaneous puberty was most frequently observed among variants located in the DBD (95.0%, 19/20), followed by the LBD (81.2%, 26/32) and the hinge region (66.7%, 18/27). In contrast, hypergonadotropic hypogonadism appeared relatively more frequently among variants affecting the hinge region (33.3%, 9/27) than among those affecting the DBD (5.0%, 1/20) and the LBD (18.8%, 6/32). In the functional subdivision of variants according to their predicted molecular consequences, spontaneous puberty was the predominant outcome across variant classes. However, hypergonadotropic hypogonadism appeared relatively more common among individuals carrying putative loss-of-function variants (23.3%, 10/43) than among those harbouring missense variants (13.9%, 5/36). In addition, when variants were analysed according to their location within the protein, missense variants were predominantly located in the DBD, whereas putative loss-of-function variants were more frequently observed in the hinge region and LBD.

Descriptive analyses of the gonadotropic axis showed elevated LH and FSH levels (mean: 24 IU/L and 72 IU/L, respectively), including among individuals with spontaneous pubertal development, while the mean testosterone level was 265.4 ng/dL (3, 14, 16, 21, 25, 26, 28, 30, 32, 34, 35, 37, 39, 46, 47, 52, 53, 54, 55, 57, 63, 64, 67, 75, 76, 82, 84, 93, 94, 96, 97, 98, 99, 102, 103, 104, 106, 111, 113, 114).

### Adrenal insufficiency

Among the 312 patients from 98 studies, only 5 patients (1.6%) presented with adrenal dysfunction and 3 (0.96%) presented with primary adrenal insufficiency (Table 2).

### Germ cell tumour

Among all 312 patients included across 98 studies, no malignant tumours were identified. However, one patient (0.32%) presented with precursor lesions of gonadal germ cell tumours, and 2 (0.64%) had gonadoblastoma. The first case involved germ cell neoplasia *in situ* (GCNIS) and involved a 13-year-old girl whose gonads were located in the abdominal and inguinal regions (39). Gonadoblastoma was identified in two individuals assigned as female: the gonads were bilaterally inguinal, and in the other, they were bilaterally abdominal (79).

### Splenic abnormalities

Across all 312 patients from 98 included studies, splenic function was reported in 21 (6.7%) individuals. Among these patients, 7 had normal splenic function, 2 presented laboratory abnormalities (thrombocytosis and Howell-Jolly bodies in blood) potentially related to splenic function, and 12 (3.8% of the total cohort) had hypoplasia or asplenism (37, 38, 47, 48, 115, 116).

### Association analyses between genotype, phenotype, and change in gender assignment

**Figure 3:**
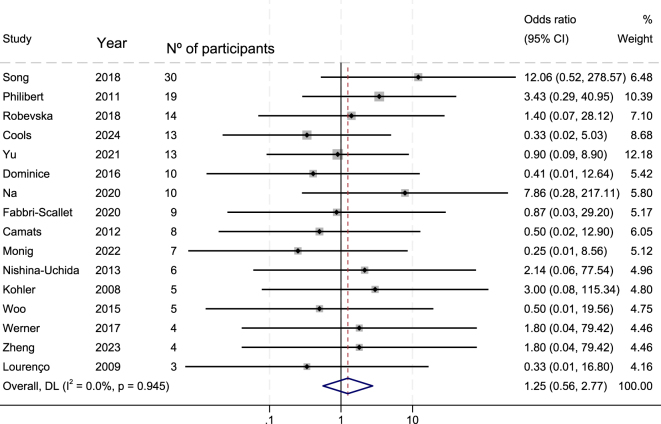
Forest plot showing the association between genotype (missense vs nonmissense variants) and the external genitalia phenotype in 46,XY DSD individuals harbouring *NR5A1* variants. Forest plot depicting the association between the *NR5A1* variant type and the external genitalia phenotype in individuals with 46,XY differences of sex development (DSD). The genotypes were categorised as missense or nonmissense variants. The effect size is expressed as odds ratios (ORs) with corresponding 95% confidence intervals (CIs), with a female external genitalia phenotype defined as the outcome and compared against an atypical external genitalia phenotype. ORs represent the odds of presenting a female external genitalia phenotype among individuals carrying nonmissense variants relative to those carrying missense variants. Individual study estimates and their relative weights are shown, together with the pooled effect derived from the meta-analysis. The horizontal lines represent 95% CIs, and the vertical line indicates the null effect (OR = 1).

**Figure 4:**
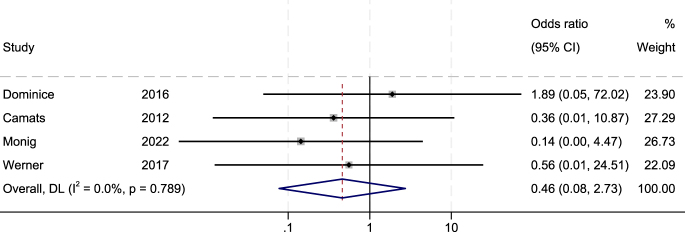
Forest plot showing the associations between the external genitalia phenotype at birth (female-like vs atypical) and change in gender assignment in 46,XY DSD individuals harbouring *NR5A1* variants. Forest plot depicting the association between the external genitalia phenotype at birth and change in gender assignment in individuals with 46,XY in differences of sex development (DSDs) carrying *NR5A1* variants. Exposure was defined as a female external genitalia phenotype at birth, whereas an atypical external genitalia phenotype was considered the reference (control) group. The outcome was a change in gender assignment during follow-up. The effect size is expressed as odds ratios (ORs) with corresponding 95% confidence intervals (CIs), representing the odds of change in gender assignment among individuals born with female external genitalia compared with those born with atypical external genitalia. Individual study estimates and their relative weights are shown, together with the pooled effect derived from the meta-analysis. The horizontal lines represent 95% CIs, and the vertical line indicates the null effect (OR = 1).

Associations between the external genital phenotype and genotype, as well as between the phenotype and changes in gender assignment, were evaluated via ORs as measures of effects. No statistically significant correlations were observed: OR = 1.25 (235 patients from 35 studies 95% CI: 0.56–2.77, *I*^2^ = 0.0%) for genotype–phenotype associations (Fig. 3) and OR = 0.46 (104 patients from 21 studies 95% CI: 0.08–2.73, *I*^2^ = 0.0%) for the association between the external genital phenotype and changes in gender assignment (Fig. 4). The OR was also determined after excluding VUS variants yielding a similar result: OR = 1.06 (170 patients from 26 studies; 95% CI: 0.49–2.33), with no statistically significant association between phenotype and genotype when considering only pathogenic and likely pathogenic variants.

The meta-regression was performed using study-level aggregated data and explores potential sources of heterogeneity across studies rather than testing associations at the individual level. The analysis revealed no significant associations since the confidence intervals included zero and a *P*-value ≥0.05, indicating that the observed differences were likely due to chance.

## Discussion

The *NR5A1* gene plays a pivotal role in early gonadal development, testicular differentiation, and steroidogenesis. *NR5A1* variants are among the most common genetic causes of 46,XY DSD, particularly in cases of partial gonadal dysgenesis, in which *NR5A1* is the most frequently implicated gene (16, 117).

### Genotype–phenotype correlation

Although *NR5A1* variants were initially reported in individuals with adrenal insufficiency, gonadal dysgenesis, and Müllerian remnants, such presentations appear to be rare. In our review, only 5 of 312 reported individuals with the 46,XY karyotype and *NR5A1* variants had impaired adrenal function, confirming that adrenal dysfunction is an uncommon feature in this condition.

The phenotypic spectrum associated with *NR5A1* variants is notably broad, ranging from complete gonadal dysgenesis with female-appearing external genitalia to milder presentations such as penoscrotal hypospadias or isolated micropenis. Importantly, a substantial proportion of individuals (*n* = 98) did not present with atypical genitalia at birth, being first evaluated in adolescence owing to signs of virilisation, delayed puberty, or primary amenorrhoea. In our dataset, atypical genitalia were reported in 85% of the cases, whereas 15% had typically female genitalia at birth.

Despite this wide range of clinical variability, the meta-analysis did not reveal a significant association between variant type (missense vs nonmissense) and the degree of genital virilisation. This observation suggests that *NR5A1*-related phenotypes may be influenced by additional genetic, epigenetic, or environmental factors beyond the primary *NR5A1* variant.

Interestingly, variant clustering was observed at positions 84 (DBD), 211 (hinge region), and 313 (LBD). Here, variant clustering refers to the descriptive, nonrandom distribution of reported variants across the gene or protein sequence and does not imply causality or functional impact. Notably, independent functional studies have demonstrated that these regions are involved in key aspects of *NR5A1* activity, including DNA binding, transcriptional regulation, and protein–protein interactions. Compared with wild-type NR5A1/SF-1, the *NR5A1* variants p.Arg84Cys and p.Tyr211ThrfsX83 have been shown to exhibit reduced DNA binding affinity and impaired transcriptional activity compared with wild-type NR5A1/SF-1 (3, 118), and the p.Arg84His variant has also been reported to disrupt transcriptional regulation (32). In addition, p.Arg313Cys was associated with decreased transactivation of the *AMH* and *CYP11A1* promoters (13). Nevertheless, the functional consequences of individual *NR5A1* variants remain incompletely understood, and further functional studies are needed to clarify their biological and clinical relevance.

### Pubertal outcome

The frequency of spontaneous puberty was 82% in our systematic review. The high rate of spontaneous puberty among 46,XY individuals harbouring *NR5A1* variants is consistent with other studies that also reported spontaneous pubertal development in most 46,XY individuals with *NR5A1* variants (113, 117). This finding contrasts with insufficient prenatal androgen production, which requires careful consideration of definitive procedures, especially genital surgeries. The near-normal testosterone levels observed during puberty in many individuals suggest that *NR5A1* function may be less critical for pubertal steroidogenesis than for fetal androgen production (15). Male sexual development involves distinct populations of Leydig cells present during fetal and postnatal life. Before birth, androgen production by fetal Leydig cells relies on a complex, compartmentalised steroidogenic pathway and differs fundamentally from that of adult Leydig cells. Unlike adult Leydig cells, which express all the enzymes required for testosterone synthesis and are luteinising hormone (LH) dependent, fetal Leydig cells lack HSD17B3 expression, relying on Sertoli cells for the final conversion of androstenedione to testosterone and maintaining relative independence from pituitary LH (119, 120).

Variants located in the hinge region of *NR5A1* were more frequently associated with hypergonadotropic hypogonadism in our analysis, with a greater proportion of loss-of-function variants observed in this domain. The hinge region connects the DBD and LBD and is important for maintaining the structural flexibility required for proper transcriptional activity of SF-1. Disruption of this region may impair transcriptional regulation and could be associated with progressive gonadal dysfunction, with a potential link to hypergonadotropic hypogonadism during adolescence or adulthood.

Although spontaneous puberty was observed in a substantial proportion of individuals, this finding should not be interpreted as evidence of normal gonadal function. In many cases, the hormonal profile was consistent with compensated hypogonadism, characterised by relatively preserved testosterone levels despite elevated gonadotropins. Our evaluation of the gonadotropic axis revealed a trend towards hypergonadotropic hypogonadism even among individuals with spontaneous pubertal development, with mean LH and FSH levels of 24 IU/L and 72 IU/L, respectively. This pattern indicates impaired Leydig and particularly Sertoli cell function, suggesting that pubertal onset may occur despite underlying gonadal dysfunction. Furthermore, Sertoli cell impairment may compromise spermatogenesis and future fertility potential. Together, these findings highlight the progressive nature of gonadal dysfunction in individuals with *NR5A1* variants and reinforce the importance of long-term endocrine and reproductive follow-up.

### Germ cell tumour

In cases of gonadal dysgenesis, assessing the extent of endogenous gonadal function allows evaluation of the potential long-term benefits and supports risk stratification of gonadal germ cell tumour risk, which, together with other conditions, is related to the degree of gonadal differentiation (121). According to our review, the risk profile of gonadal germ cell tumours in individuals with *NR5A1* variants is low. However, the degree of virilisation may influence this risk, as precursor lesions were found exclusively in individuals raised as females. These findings emphasise the need for individualised risk assessment and ongoing gonadal surveillance in all patients.

### Splenic involvement

Although *NR5A1* is primarily recognised for its role in gonadal and adrenal development, extragonadal manifestations such as impaired splenic development/function have been reported (37, 38, 47, 48, 115, 116). In our synthesis, splenic function was documented in only 6.7% of individuals, yet 3.8% of the entire cohort had splenic hypoplasia or asplenia, highlighting both clinical relevance and substantial underreporting in the literature.

### Sex assignment

Despite the discrepancy between the number of individuals with atypical genitalia (*n* = 252) and those with female-appearing genitalia (*n* = 60), sex assignment at birth was relatively balanced, with 54% raised as female and 46% as male. This finding suggests that external genital appearance alone was likely not the major determinant of sex assignment decisions in individuals with *NR5A1* variants. Gender rearing is a crucial topic in the management of individuals with *NR5A1* variants. Boys with these variants are highly likely to undergo spontaneous puberty (15, 98, 122) and most 46,XY individuals assigned female at birth will exhibit progressive virilisation if the gonads are preserved (15). These findings indicate that pubertal virilisation is driven primarily by the presence of functional testosterone-producing tissue, independent of the sex of rearing.

Although a change in gender assignment is relatively uncommon (10%), it is noteworthy that no cases of male-to-female transition have been reported in the literature thus far.

### Study limitations

This review has several limitations. The data were derived primarily from observational studies and case reports, many of which included incomplete or selectively reported information. Consequently, the overall quality of evidence was very low, with wide confidence intervals and small sample sizes, and the findings did not meet criteria for upgrading the level of evidence (24).

The binary classification of the external genital phenotype into ‘female-like’ and ‘atypical’, although methodologically pragmatic, may have reduced phenotypic granularity, as most primary studies lacked sufficient anatomical detail to apply standardised scoring systems retrospectively. Further stratification would likely have resulted in small cell counts and increased the risk of statistical instability and sparse-data bias. In addition, limited sample sizes – particularly in subgroup analyses and for less frequent outcomes such as gender transition or adrenal insufficiency – reduced statistical power to detect meaningful associations. Correlations involving genotype and pubertal outcomes, as well as phenotype and changes in gender assignment, could not be adequately assessed due to insufficient stratified data across studies. Moreover, pubertal outcomes were frequently underreported, highlighting the need for long-term follow-up beyond childhood and adolescence.

Despite these limitations, this systematic review provides the most comprehensive synthesis to date of the phenotypic variability and clinical implications of *NR5A1* variants in 46,XY individuals, offering valuable guidance for diagnostic and therapeutic decision-making.

## Conclusion

Individuals with *NR5A1* variants exhibit marked phenotypic variability, reflecting the complex regulation of gonadal and adrenal development. This systematic review provides a valuable and comprehensive synthesis of the current understanding of the genotype–phenotype spectrum in 46,XY DSD individuals with *NR5A1* defects.

These findings support the need for individualised and cautious management. Although spontaneous pubertal development was frequently observed, and the reported incidence of gonadal germ cell tumours appeared low, these findings should be interpreted with caution, as they may be influenced by reporting bias and limited follow-up inherent to the available evidence. Accordingly, irreversible procedures, such as gonadectomy or genital surgery, may be deferred when clinically appropriate until pubertal potential and gender identity can be more clearly assessed.

Long-term follow-up should include hormonal monitoring, gonadal imaging when indicated, and psychological support, ensuring truly personalised care across development.

Ultimately, *NR5A1*-related DSD exemplifies the shift from anatomy-based decisions at birth towards lifespan-oriented management, integrating genetic, endocrine, and psychosocial dimensions.

## Supplementary materials



## Declaration of interest

The authors declare that there is no conflict of interest that could be perceived as prejudicing the impartiality of the work reported.

## Funding

This study received support from the São Paulo State Research Support Foundation (FAPESP; 2019/26780-9).

## Data availability

Original data generated and analysed during this study are included in this published article or in the data repositories listed in the references.
