# Supplementary material for: Spectrum and genotype–phenotype correlation of NR5A1 variants in 46,XY DSD: a systematic review and meta-analysis
Source: Endocr Connect. 2026 May 6;15(5):e250783. doi: 10.1530/EC-25-0783 (PMC13178433; doi:10.1530/EC-25-0783)
Supplement: Supplementary file 1 [file supplementary_materials.pdf]

Spectrum of *NR5A1* Variants in 46, XY DSD: Systematic Review and Meta-analysis

Renata Thomazini Dallago [1,2], Rafael Loch Batista[1,2], Sorahia Domenice[1,2], Vania dos Santos Nunes-Nogueira[3] and Berenice Bilharinho Mendonca[1,2]

1 - Developmental Endocrinology Unit, Endocrinology Division, Internal Medicine Department, Medical School, University of São Paulo (USP), São Paulo, SP, Brazil

2 - Hormone and Molecular Genetics Laboratory (LIM/42), Hospital das Clínicas da Universidade de São Paulo, São Paulo, SP, Brazil

3- São Paulo State University (UNESP), Medical School

ORCID numbers: 0000-0002-0754-0718 (R. T. Dallago), 0000-0002-5461-0301 (R. L. Batista), 0000-0002-4883-7630 (S. Domenice), 0000-0001-9316-4167 (V. S. Nunes-Nogueira), 0000-0003-1762-1084 (B. B. Mendonca)

Corresponding author:
Renata Thomazini Dallago

Address: Avenida Dr Arnaldo, 455, Faculdade de Medicina da Universidade de São Paulo (USP), São Paulo, SP, Brazil, Zip Code: 01246-903, Tel: +55 (45) 99123-1989

E-mail: renatatdallago@usp.br

Keywords: Disorders of sex development; 46,XY DSD; Gonadal dysgenesis; Steroidogenic fator 1; SF-1 protein.

Disclosure: The authors declare no conflicts of interest.

Table 1. The index term and synonyms used in the database Search ---3

Table 2. The risk of bias in accordance with the Joanna Briggs Institute checklist for case series -----------------------------------6

Table 3. The excluded studies with the reasons ---8

Figure 1. The Overall Frequency of Missense Variants ---9

Figure 2. The Overall Frequency of Nonsense Variants --10

Figure 3. The Overall Frequency of Atypical Genitalia ---11

Figure 4. The Meta-regression of Atypical Genitalia using the Nonsense Variant as Covariate --------------------------------------12

Figure 5. The Overall Frequency of Female-like Genitalia ---13

Figure 6. The Meta-regression of Spontaneous Puberty using the Missense Variant as Covariate -----------------------------------14

Figure 7. The Meta-regression of Gonadectomy using the Missense Variants as Covariate ---14

Figure 8. The Overall Frequency of Gonadectomy Prepubertal --15

Figure 9. The Overall Frequency of Hypergonadotropic Hypogonadism ---16

Figure 10. The Overall Frequency of Spontaneous Puberty ---17

Figure 11. The Overall Frequency of Gender Change ---18

- **Supplementary material**

Table 1. The index term and synonyms used in the database search.

Pubmed		
Mesh term	Entry terms	Results
#1 Disorders of Sex Development	"Disorders of Sex Development" [Mesh] OR (Sex Development Disorders) OR (Sex Development Disorder) OR (Disorders of Sexual Development) OR (Sexual Development Disorders) OR (Sexual Development Disorder) OR (Ambiguous Genitalia) OR (Genitalia, Ambiguous) OR (Genital Ambiguity) OR (Ambiguities, Genital) OR (Ambiguity, Genital) OR (Genital Ambiguities) OR (Intersex Conditions) OR (Condition, Intersex) OR (Conditions, Intersex) OR (Intersex Condition) OR (Pseudohermaphroditism) OR (Sex Differentiation Disorders) OR (Differentiation Disorder, Sex) OR (Differentiation Disorders, Sex) OR (Disorder, Sex Differentiation) OR (Disorders, Sex Differentiation) OR (Sex Differentiation Disorder) OR (Sexual Differentiation Disorders) OR (Differentiation Disorder, Sexual) OR (Differentiation Disorders, Sexual) OR (Disorder, Sex Differentiation) OR (Disorders, Sex Differentiation) OR (Sexual Differentiation Disorder) OR (Hermaphroditism) OR (Intersexuality) OR (Intersexualities)	68,375
#2 Disorder of Sex Development, 46,XY	"Disorder of Sex Development, 46,XY" [Mesh] OR (46,XY Disorders of Sex Development) OR (46,XY DSD) OR (46,XY DSDs) OR (DSD, 46,XY) OR (DSDs, 46,XY) OR (46, XY Disorders of Sex Development) OR (46, XY DSD) OR (46,XY Sex Reversal 3) OR (46,XY Sex Reversal, Partial or Complete, NR5A1-Related) OR (Sex Reversal, XY, With Or Without Adrenal Failure) OR (46,XY Gonadal Dysgenesis, Complete or Partial, With or Without Adrenal Failure) OR (Male Pseudohermaphroditism) OR (Male Pseudohermaphroditisms) OR (Pseudohermaphroditism, Male) OR (Pseudohermaphroditisms, Male)	574,546
#3 Gonadal Dysgenesis, 46,XY	"Gonadal Dysgenesis, 46,XY" [Mesh] OR (Sex Reversal, Gonadal, 46, XY) OR (Gonadal Dysgenesis, 46, XY) OR (46, XY Gonadal Dysgenesis) OR (46, XY Gonadal Sex Reversal) OR (Swyer Syndrome) OR (Syndrome, Swyer) OR (Pure Gonadal Dysgenesis 46,XY) OR (46,XY Complete Gonadal Dysgenesis) OR (XY Pure Gonadal Dysgenesis) OR (Complete Gonadal Dysgenesis, 46, XY) OR (Pure Gonadal Dysgenesis, 46, XY)	2,007
#4 Steroidogenic Factor 1	"Steroidogenic Factor 1" [Mesh] OR (Ad4-Binding Protein) OR (Ad4 Binding Protein) OR (Adrenal 4 Binding Protein) OR (AD4BP Protein) OR (Fushi Tarazu Factor Homolog 1) OR (NR5A1 Protein) OR (Nuclear Receptor 5A1	2,165

	Protein) OR (FTZF1 Protein) OR (SF-1 Transcription Factor) OR (Transcription Factor, SF-1) OR (Steroid Hormone Receptor Ad4BP)	
#5 SF1 protein, human [Supplementary Concept]	"SF1 protein, human" [Supplementary Concept] OR (splicing factor 1 protein, human) OR (zinc finger protein 162, human) OR (ZFM1 protein, human) OR (ZNF162 protein, human)	101
#6 NR5A1 protein, human [Supplementary Concept]	"NR5A1 protein, human" [Supplementary Concept] OR (FTZF1 protein, human) OR (steroidogenic factor 1, human) receptor subfamily 5, group A, member 1 protein, human) OR (AD4BP protein, human) OR (fushi tarazu factor (Dro homolog 1 protein, human)	490
Total = (#1 OR #2 OR #3) AND (#4 OR #5 OR #6)	"Disorders of Sex Development"[Mesh] OR (Sex Development Disorders) OR (Sex Development Disorder) OR (Disorders of Sexual Development) OR (Sexual Development Disorders) OR (Sexual Development Disorder) OR (Ambiguous Genitalia) OR (Genitalia, Ambiguous) OR (Genital Ambiguity) OR (Ambiguities, Genital) OR (Ambiguity, Genital) OR (Genital Ambiguities) OR (Intersex Conditions) OR (Condition, Intersex) OR (Conditions, Intersex) OR (Intersex Condition) OR (Pseudohermaphroditism) OR (Sex Differentiation Disorders) OR (Differentiation Disorder, Sex) OR (Differentiation Disorders, Sex) OR (Disorder, Sex Differentiation) OR (Disorders, Sex Differentiation) OR (Sex Differentiation Disorder) OR (Sexual Differentiation Disorders) OR (Differentiation Disorder, Sexual) OR (Differentiation Disorders, Sexual) OR (Disorder, Sex Differentiation) OR (Disorders, Sex Differentiation) OR (Sexual Differentiation Disorder) OR (Hermaphroditism) OR (Intersexuality) OR (Intersexualities) OR "Disorder of Sex Development, 46,XY"[Mesh] OR (46,XY Disorders of Sex Development) OR (46,XY DSD) OR (46,XY DSDs) OR (DSD, 46,XY) OR (DSDs, 46,XY) OR (46, XY Disorders of Sex Development) OR (46, XY DSD) OR (46,XY Sex Reversal 3) OR (46,XY Sex Reversal, Partial or Complete, NR5A1-Related) OR (Sex Reversal, XY, With Or Without Adrenal Failure) OR (46,XY Gonadal Dysgenesis, Complete or Partial, With or Without Adrenal Failure) OR (Male Pseudohermaphroditism) OR (Male Pseudohermaphroditisms) OR (Pseudohermaphroditism, Male) OR (Pseudohermaphroditisms, Male) OR "Gonadal Dysgenesis, 46,XY"[Mesh] OR (Sex Reversal, Gonadal, 46, XY) OR (Gonadal Dysgenesis, 46, XY) OR (46, XY Gonadal Dysgenesis) OR (46, XY Gonadal Sex Reversal) OR (Swyer Syndrome) OR (Syndrome, Swyer) OR (Pure Gonadal Dysgenesis 46,XY) OR (46,XY Complete Gonadal Dysgenesis) OR (XY Pure Gonadal Dysgenesis) OR (Complete Gonadal Dysgenesis, 46, XY) OR (Pure Gonadal Dysgenesis, 46, XY) AND "Steroidogenic Factor 1"[Mesh] OR (Ad4-Binding Protein) OR (Ad4 Binding Protein) OR (Adrenal 4 Binding Protein) OR (AD4BP Protein) OR (Fushi Tarazu Factor Homolog 1) OR (NR5A1 Protein) OR (Nuclear Receptor 5A1 Protein) OR (FTZF1 Protein) OR (SF-1 Transcription Factor) OR (Transcription Factor, SF-1) OR (Steroid Hormone Receptor Ad4BP) OR "SF1 protein, human" [Supplementary Concept] OR (splicing factor 1 protein, human) OR (zinc finger protein 162, human) OR (ZFM1 protein, human)	351

	OR (ZNF162 protein, human) OR "NR5A1 protein, human" [Supplementary Concept] OR (FTZF1 protein, human) OR (steroidogenic factor 1, human) OR (nuclear receptor subfamily 5, group A, member 1 protein, human) OR (AD4BP protein, human) OR (fushi tarazu factor (Drosophila) homolog 1 protein, human)	

**Embase**		
Emtree	Entry terms	Results
#1 'disorder of sex development'/exp	'disorder of sex development'/exp<br>OR 'disorder of sex development'/exp OR '46, XX disorders of sex development' OR '46, XX testicular disorders of sex development' OR '46, XY disorders of sex development' OR '46, XX DSD' OR '46, XY disorder of sex development' OR '46, XY DSD' OR 'difference of sex development' OR 'differences of sex development' OR 'disorder of sex development, 46, XY' OR 'disorder of sex differentiation' OR 'disorder of sexual development' OR 'disorder of sexual differentiation' OR 'disorders of sex development' OR 'disorders of sex differentiation' OR 'disorders of sexual development' OR 'disorders of sexual differentiation' OR 'divergence of sex development' OR 'divergences of sex development' OR 'DSD (disorder of sex development)' OR 'intersexualism' OR 'intersexuality' OR 'ovotesticular disorders of sex development' OR 'sex chromosome disorder of sex development' OR 'sex chromosome disorders of sex development' OR 'sex development disorder' OR 'sex development disorders' OR 'sex differentiation disorder' OR 'sex differentiation disorders' OR 'sex differentiation disturbance' OR 'sexual development disorder' OR 'sexual development disorders' OR 'sexual differentiation disorder' OR 'sexual differentiation disorders' OR 'disorder of sex development'	58,005
#2 'XY gonadal dysgenesis'/exp	'XY gonadal dysgenesis'/exp<br>OR '46, XY CGD' OR '46, XY complete gonadal dysgenesis' OR '46, XY gonadal dysgenesis' OR '46, XY pure gonadal dysgenesis' OR 'complete gonadal dysgenesis, 46, XY' OR 'gonadal dysgenesis, 46, XY' OR 'gonadal dysgenesis, pure 46xy' OR 'pure gonadal dysgenesis 46, XY' OR 'Swyer syndrome' OR 'Swyer`s syndrome' OR 'XY pure gonadal dysgenesis' OR 'XY gonadal dysgenesis'	454
#3 'steroidogenic factor 1'/exp	'steroidogenic factor 1'/exp<br>OR 'adrenal 4 binding protein' OR 'steroidogenic factor 1'	2,166
#4 candidate term ‘NR5A1 protein’/exp	‘NR5A1 protein’/exp OR 'nr5a1 gene' OR 'nr5a1 protein human' OR 'ftz f1 gene' OR 'sf 1 gene'	303
Total = #1 OR #2 AND #3 OR #4		422

Table 2. The risk of bias in accordance with the Joanna Briggs Institute checklist for case series

Author (year)	Clear inclusion criteria	Diagnostic criteria stated	Valid methods used for identification of the condition	Consecutive and complete inclusion of participants	Complete reporting of clinical information	Complete reporting of outcomes	Complete reporting of demographics of participants and site/clinic(s)	Apropriate statistical analyses	Risk of bias classification (low, moderate and high)
Allali (2011)	Y	Y	Y	Y	Y	N	Y	NA	Low
Baetens (2014)	Y	Y	Y	Y	Y	NA	Y	NA	Low
Buonocore (2019)	Y	Y	Y	Y	Y	Y	Y	Y	Low
Camats (2012)	Y	Y	Y	N	Y	N	Y	NA	Moderate
Ciaccio (2012)	Y	Y	Y	Y	Y	Y	Y	NA	Low
Cools (2024)	Y	Y	Y	Y	Y	Y	Y	Y	Low
Del Gobbo (2024)	Y	Y	Y	U	N	N	Y	NA	Moderate
Domenice (2016)	Y	Y	Y	Y	Y	Y	Y	NA	Low
Eggers (2015)	Y	Y	Y	Y	Y	N	N	NA	Moderate
Fabbri (2016)	Y	Y	Y	Y	Y	Y	N	NA	Low
Fabbri-Scallet (2022)	Y	Y	Y	Y	Y	Y	Y	NA	Low
Fabbri-Scallet (2020)	Y	Y	Y	Y	Y	Y	Y	NA	Low
Fabbri-Scallet (2018)	Y	Y	Y	Y	Y	Y	Y	NA	Low
Faienza (2020)	Y	Y	Y	Y	Y	N	Y	NA	Low
Hussain (2016)	Y	Y	Y	Y	Y	N	Y	NA	Low
Kohler (2008)	Y	Y	Y	Y	Y	Y	Y	NA	Low
Kohler (2009)	Y	Y	Y	Y	Y	Y	Y	NA	Low
Lin Lin (2007)	Y	Y	Y	Y	Y	Y	Y	NA	Low
Lourenço (2009)	Y	Y	Y	Y	Y	Y	Y	NA	Low

Table 2 continuation

Malikova (2014)	Y	Y	Y	Y	Y	N	Y	NA	Low
Monig (2022)	Y	Y	Y	Y	Y	Y	Y	NA	Low
Na (2020)	Y	Y	Y	Y	Y	N	Y	NA	Low
Nishina-Uchida (2013)	Y	Y	Y	Y	Y	N	Y	NA	Low
Ochoa (2021)	Y	Y	Y	Y	Y	N	Y	NA	Low
Philibert (2011)	Y	Y	Y	N	N	N	Y	NA	Moderate
Robevska (2018)	Y	Y	Y	Y	Y	Y	Y	NA	Low
Rocca (2018)	Y	Y	Y	Y	Y	Y	Y	NA	Low
Song (2018)	Y	Y	Y	Y	Y	Y	Y	Y	Low
Sudhakar (2019)	Y	Y	Y	Y	N	N	Y	NA	Moderate
Tantawy (2014)	Y	Y	Y	Y	Y	Y	Y	NA	Low
Warman (2011)	Y	Y	Y	Y	Y	Y	Y	NA	Low
Werner (2017)	Y	Y	Y	Y	Y	Y	Y	NA	Low
Woo (2015)	Y	Y	Y	Y	Y	N	Y	NA	Low
Yu (2018)	Y	Y	Y	Y	Y	N	Y	NA	Low
Zheng (2023)	Y	Y	Y	Y	Y	N	Y	NA	Low

Legenda: The qualitative classification was carried out according to the Joanna Briggs Institute checklist for case series: low risk if it met more than 70% of the criteria, moderate between 50-69% and high if less than 50%. Y = yes; N = no; U = nuclear; NA = not aplicable.

Table 3. The excluded studies with the reasons

Reason	Author (year)				
Conference abstract	Abdulhadi-Atwan (2018)<br>Achermann (2014)<br>Aghayev (2018)<br>Alimussina (2019)<br>Alimussina (2021)<br>Anik (2015)<br>Arslan (2021)<br>Astudillo (2016)<br>Baldazzi (2011)<br>Banne (2012)<br>Bashamboo (2017)<br>Bellizzi (2010)<br>Boudjemaa (2010)<br>Camats (2017)<br>Campos Fabbri (2016)	Chauhan (2015)<br>Ciaccio (2011)<br>Cuccaro (2018)<br>Dallago (2023)<br>De Lapiscina (2022)<br>Denzer (2018)<br>Dillies (2016)<br>Domenice (2004)<br>Elzenaty (2023)<br>Fabbri (2016)<br>Franklin (2022)<br>Gaisl (2019)<br>Garrido (2013)<br>Gay (2016)<br>Gomes (2017)<br>Gomes (2018)	Gomes (2018)<br>Gomes (2019)<br>Grijp (2021)<br>Hooi (2022)<br>Hoppmann (2018)<br>Improda (2013)<br>Kalfa (2022)<br>Kalinchenko (2017)<br>Kalinchenko (2019)<br>Kalinchenko (2020)<br>Kempers (2018)<br>Kim (2016)<br>Kim (2023)<br>Knoll (2020)<br>Kouri (2021)<br>Kouri (2022)<br>Kouri (2023)	Ladjouze (2011)<br>Ladjouze (2016)<br>Lapiscina (2023)<br>Livshits (2019)<br>Livshyts (2017)<br>Ma (2013)<br>Machado (2010)<br>Maciel-Guerra (2017)<br>Maciel-Guerra (2018)<br>Martinez (2015)<br>McGown (2015)<br>Nikitina (2021)<br>Ning (2017)<br>Petroli (2016)<br>Pian (2022)<br>Poyrazoglu (2019)<br>Qiao (2020)	Raafat (2023)<br>Raygorodskaya (2016)<br>Rosenheck (2021)<br>Shcherbak (2017)<br>Shcherbak (2018)<br>Shcherbak (2019)<br>Siklar (2013)<br>Sinha (2011)<br>Syryn (2023)<br>Tantawy (2014)<br>Teoli (2022)<br>Touzon (2018)<br>Touzon (2019)<br>Vela (2018)<br>Yamaguchi (2017)<br>Yongchuan (2020)<br>Yu (2022)
Benign variants	AvRuskin (2004)	Lapiscina (2023)	Paliwal (2011)	Wada (2005)	
Lack of phenotype or *NR5A1* variant information	AboElella (2015)<br>Ata (2021)<br>Bashamboo (2010)<br>Buonocore (2021)	Camats (2014)<br>Chen (2021)<br>Eggers (2016)<br>Galada (2014)	Gomes (2022)<br>Hughes (2019)<br>Knower (2011)<br>Kouri (2024)	Lim (1998)<br>Rehkamper (2018)<br>Shojaei (2017)<br>Wei (2023)	Yu (2021)<br>Zhang (2024)<br>Sreenivasan (2022)<br>Wang (2018)
Association with other conditions	Sreenivasan (2022)	Schlaubitz (2007)	Schteingart (2019)		
Overlapping cases	Andrade (2014)<br>Camats (2018)	Fabbri (2014)<br>Gomes (2018)	Harrison (2014)<br>Lin (2007)<br>Lin Lin (2006)	Mazen (2021)<br>Philibert (2010)<br>Philibert (2011)	Sreenivasan (2018)<br>Tremblay (2003)<br>Yu (2021)
Literature review	Achermann (2005)	Camats (2020)	Elzenaty (2024)	Nagaraja (2019)	

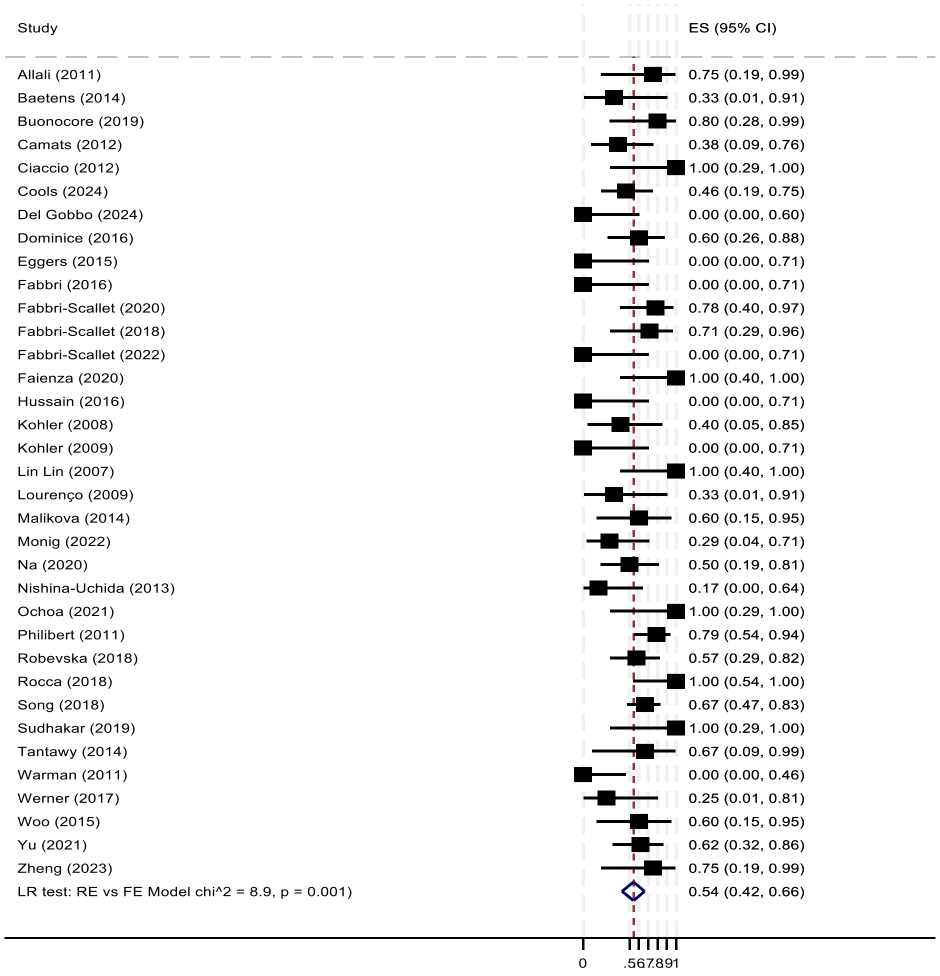

Figure 1. The Overall Frequency of Missense Mutation

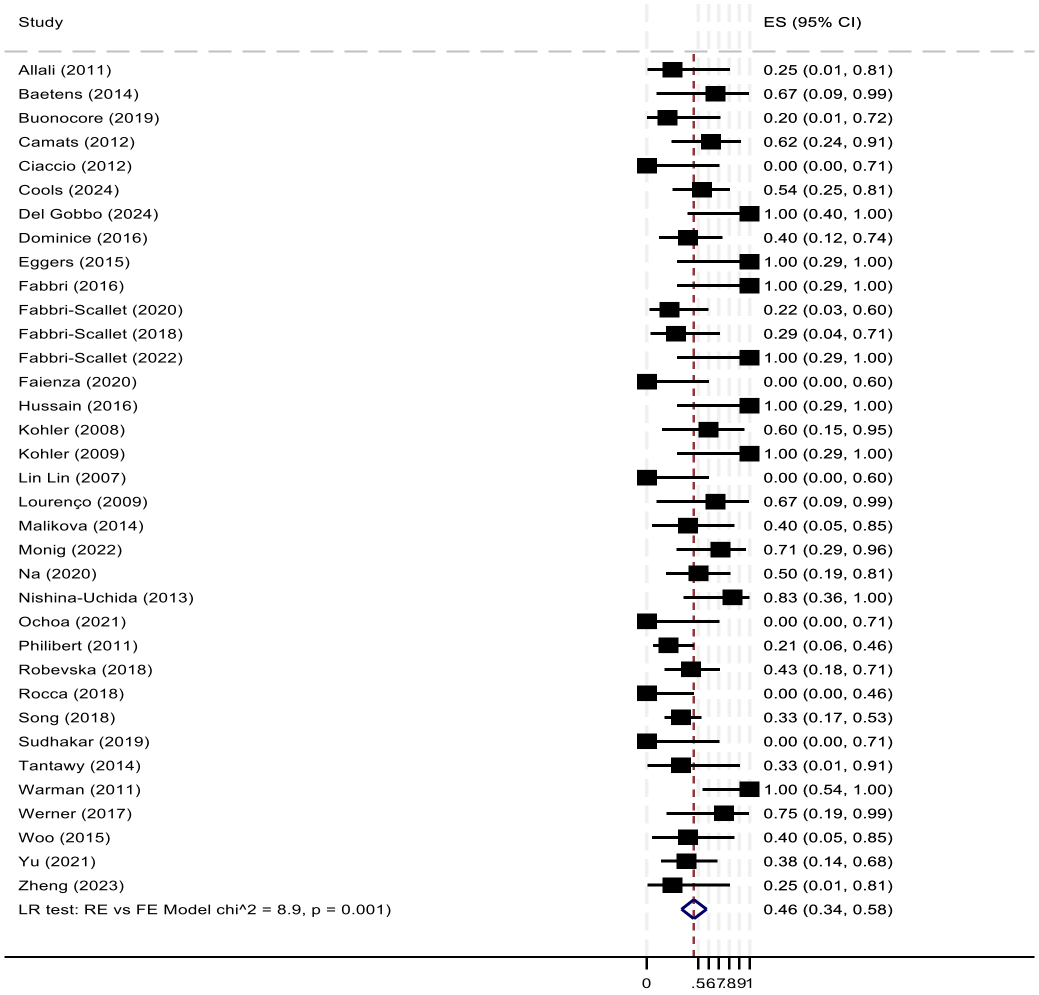

Figure 2. The Overall Frequency of Nonsense Mutation

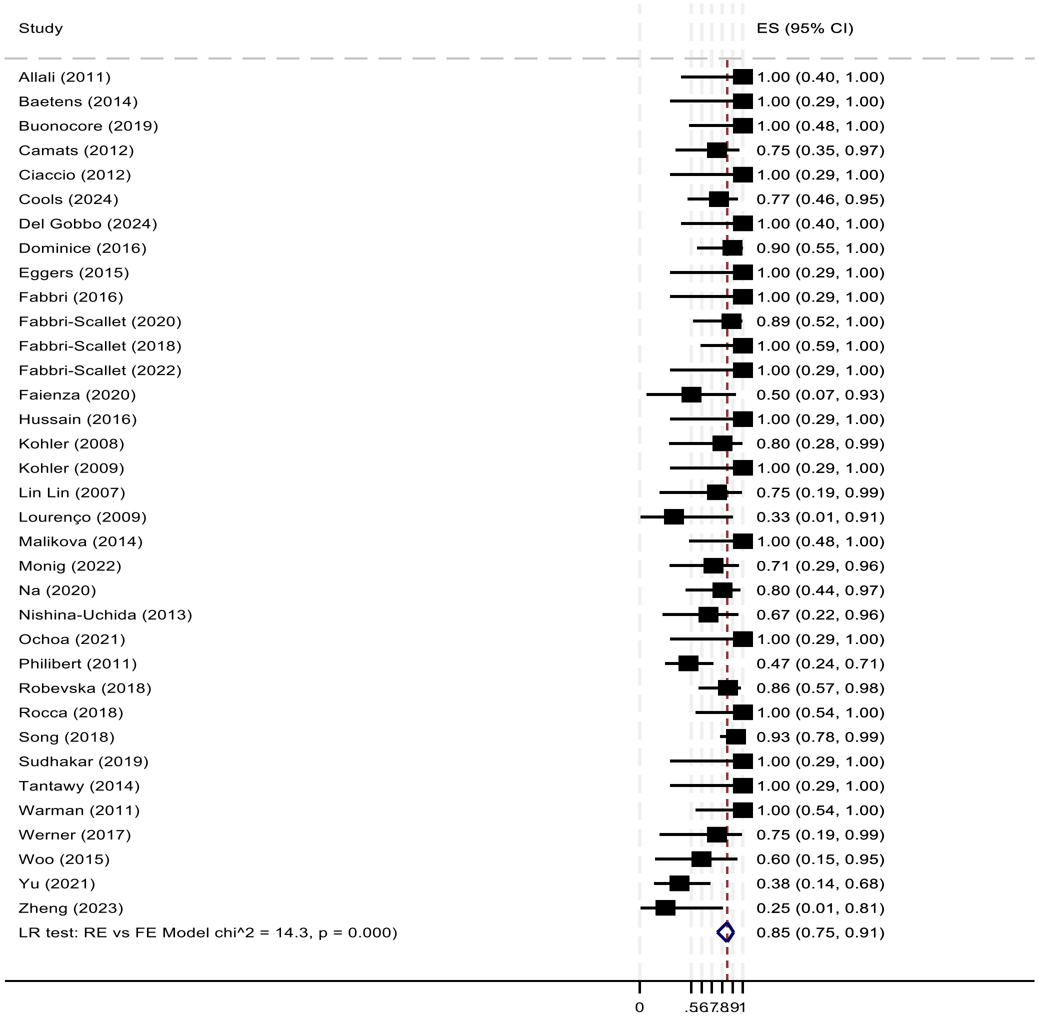

Figure 3. The Overall Frequency of Atypical Genitalia

```
.
. gen log_seES= (lnub- lnlb)/(2*invnormal(0.975))

. metareg   logES   nnonsense , wsse ( log_seES)
numerical derivatives are approximate
nearby values are missing
numerical derivatives are approximate
nearby values are missing

Meta-regression                                       Number of obs  =      35
REML estimate of between-study variance               tau2           =       0
% residual variation due to heterogeneity             I-squared_res  =   0.00%
Proportion of between-study variance explained        Adj R-squared  =      .%
With Knapp-Hartung modification
------------------------------------------------------------------------------
       logES | Coefficient  Std. err.      t    P>|t|     [95% conf. interval]
-------------+----------------------------------------------------------------
   nnonsense |  -.0024467   .0094513    -0.26   0.797    -.0216755    .0167821
       _cons |   -.086783   .0603482    -1.44   0.160    -.2095624    .0359963
------------------------------------------------------------------------------
```

Figure 4. The Meta-regression of Atypical Genitalia using the Nonsense Variants as Covariate.

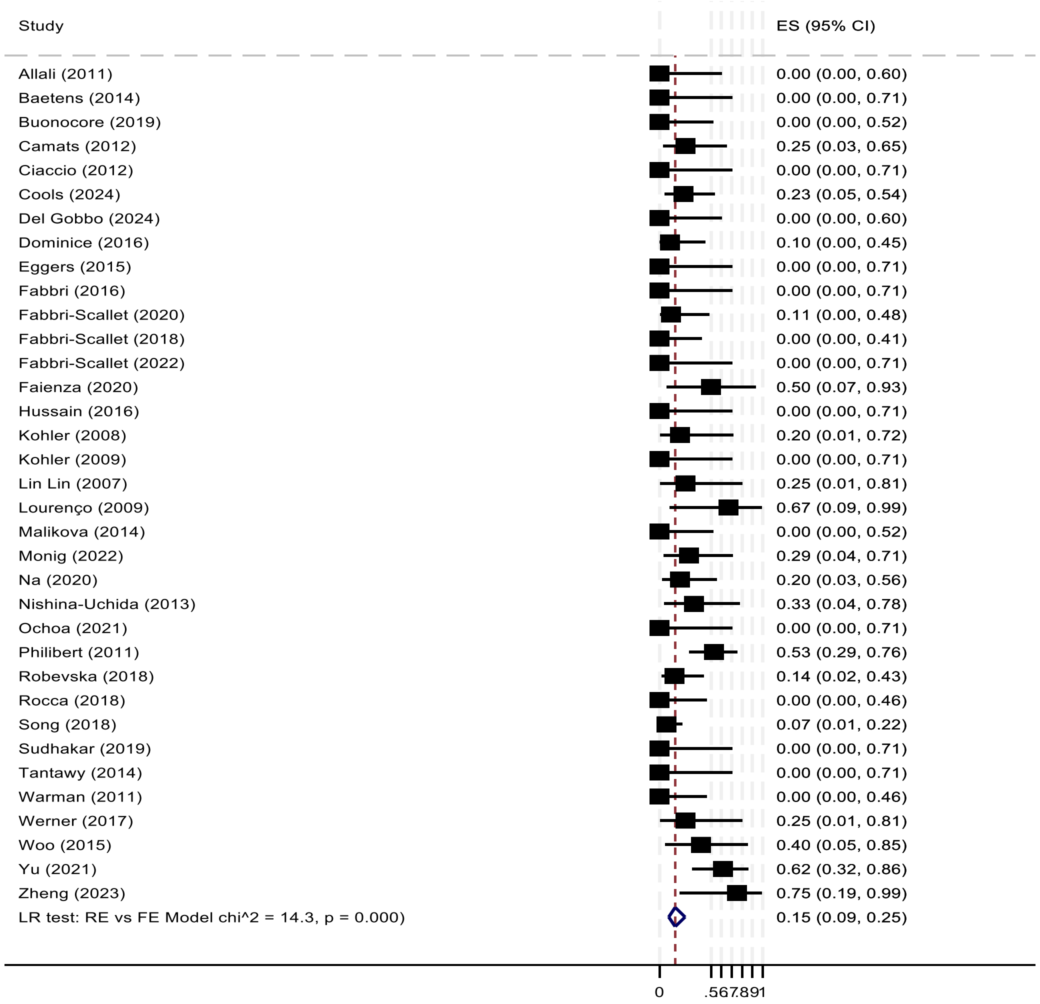

Figure 5. The Overall Frequency of Female-like Genitalia

```
. metareg   logES   nmissense, wsse ( log_seES)

Meta-regression                                      Number of obs  =      18
REML estimate of between-study variance              tau2           =       0
% residual variation due to heterogeneity            I-squared_res  =   0.00%
Proportion of between-study variance explained       Adj R-squared  =      .%
With Knapp-Hartung modification

------------------------------------------------------------------------------
       logES | Coefficient  Std. err.      t    P>|t|     [95% conf. interval]
-------------+----------------------------------------------------------------
   nmissense |   .0045032    .035644     0.13   0.901    -.0710587    .0800651
       _cons |  -.0846602   .1514421    -0.56   0.584    -.4057032    .2363828
```

Figure 6. The Meta-regression of Spontaneous Puberty using the Missense Variants as Covariate.

```
. metareg   logES   nmissense , wsse ( log_seES)

Meta-regression                                      Number of obs  =       8
REML estimate of between-study variance              tau2           = .007317
% residual variation due to heterogeneity            I-squared_res  =   0.00%
Proportion of between-study variance explained       Adj R-squared  =      .%
With Knapp-Hartung modification

------------------------------------------------------------------------------
       logES | Coefficient  Std. err.      t    P>|t|     [95% conf. interval]
-------------+----------------------------------------------------------------
   nmissense |  -.0172824   .0557622    -0.31   0.767    -.1537275    .1191627
       _cons |  -.0844675   .1984504    -0.43   0.685    -.5700581    .4011231
------------------------------------------------------------------------------
```

Figure 7. The Meta-regression of Gonadectomy using the Missense Variants as Covariate.

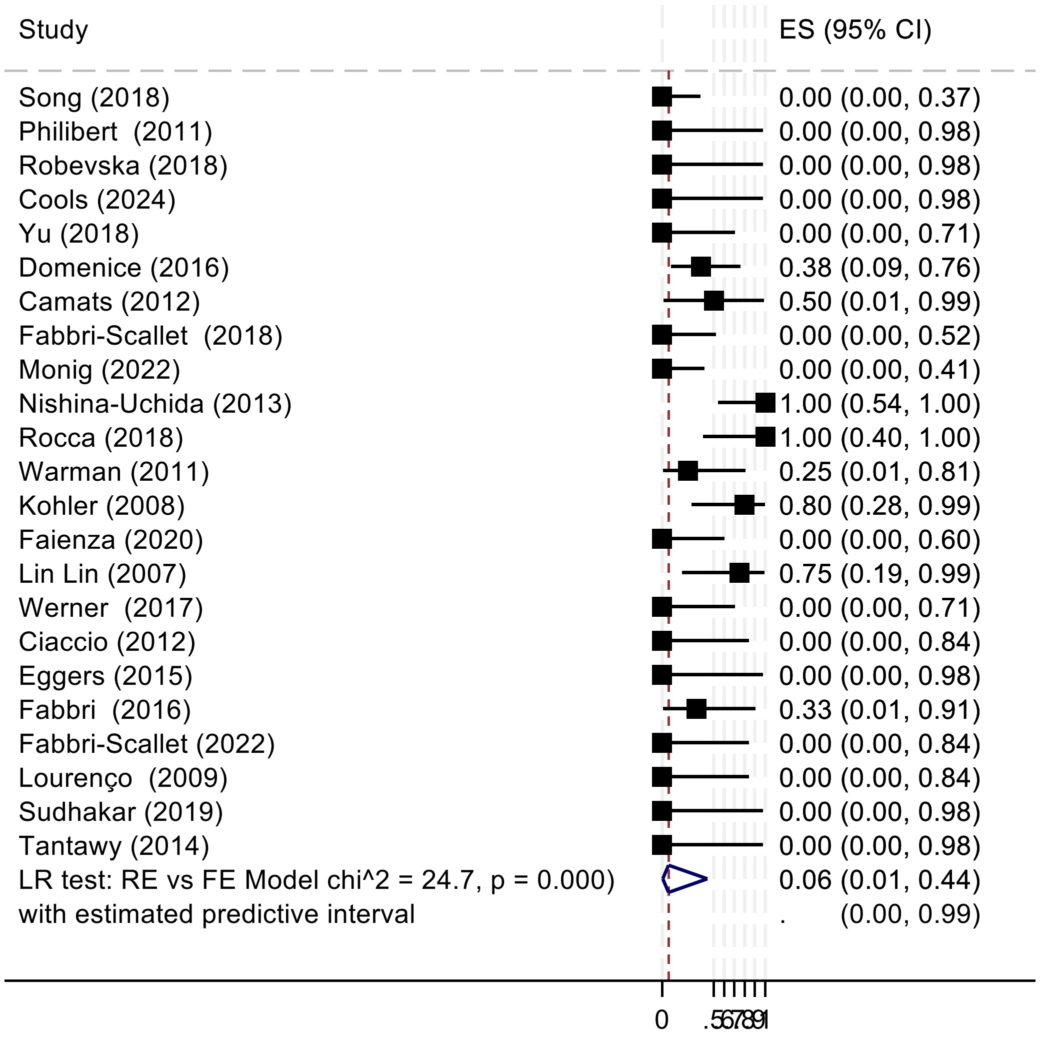

Figure 8. The Overall Frequency of Gonadectomy Prepubertal.

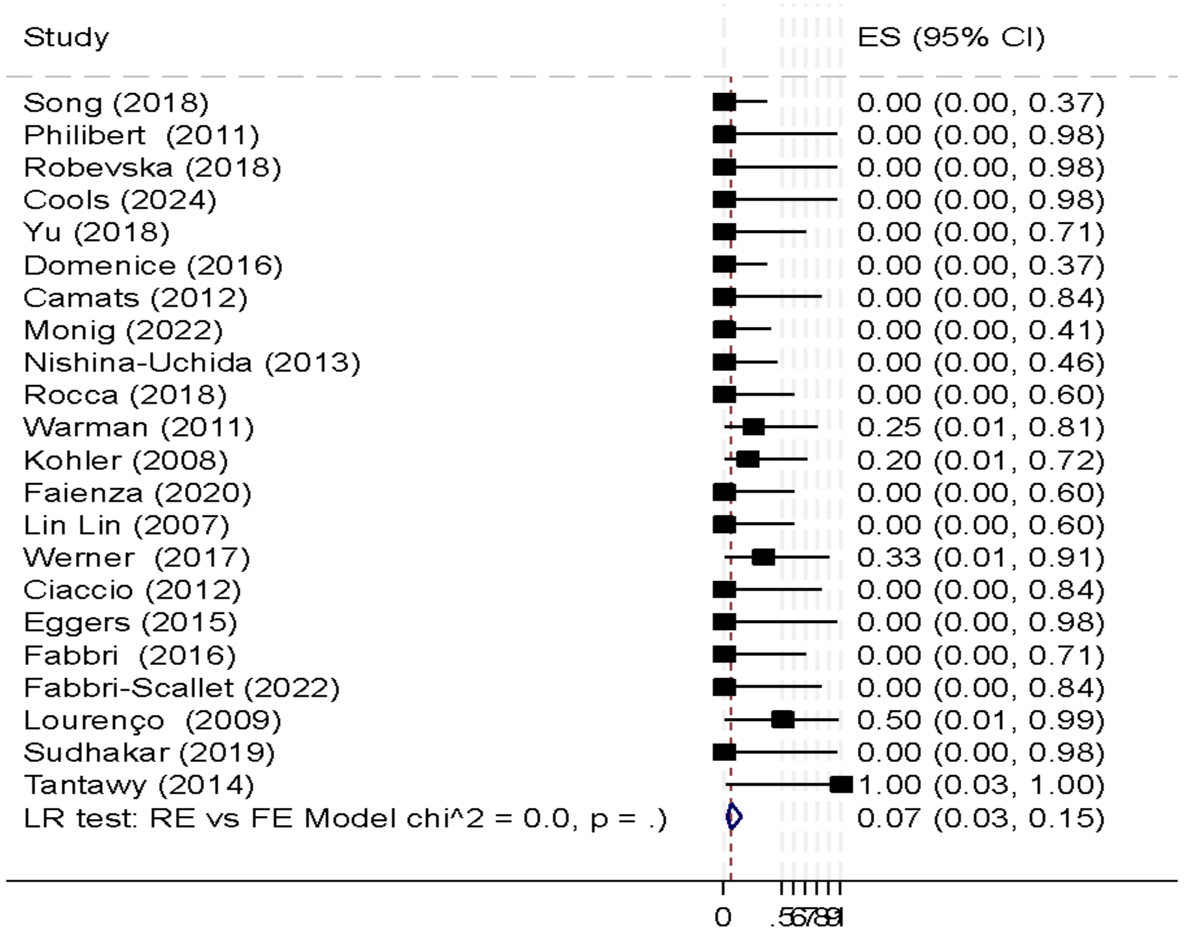

Figure 9. The Overall Frequency of Hypergonadotropic Hypogonadism

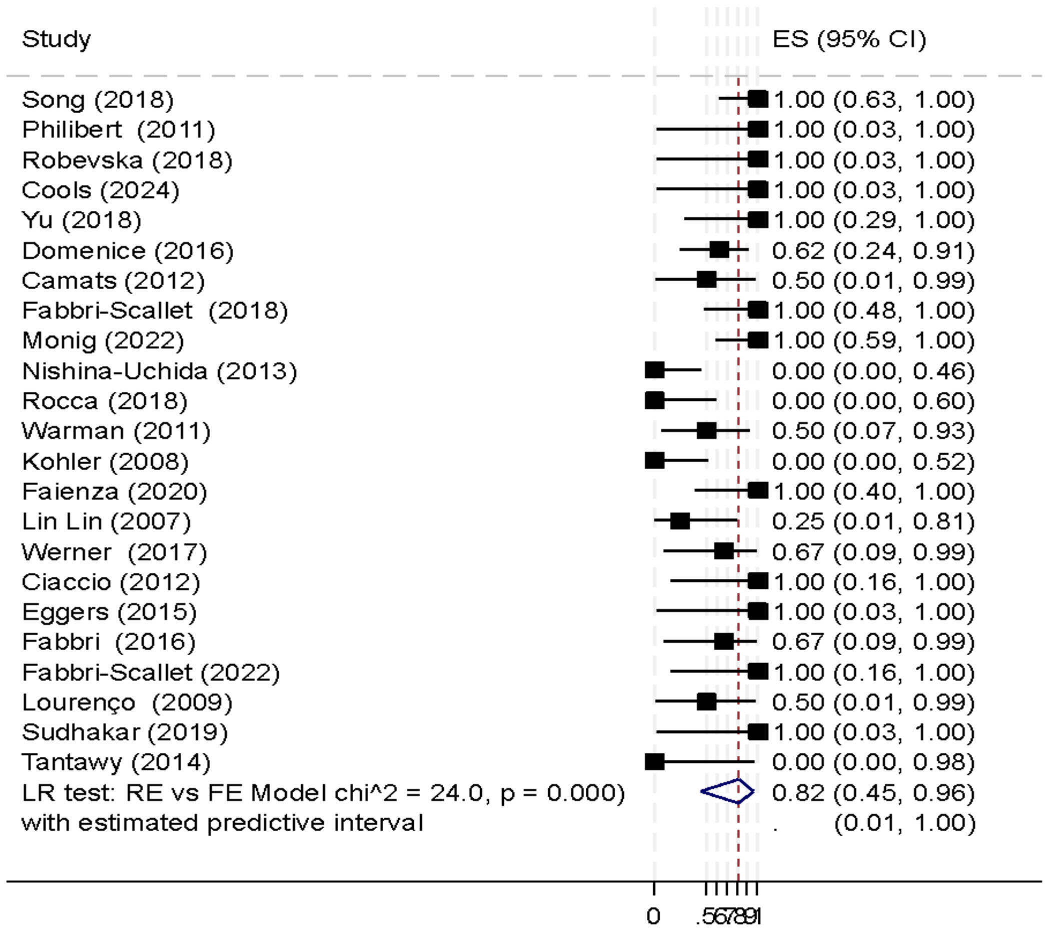

Figure 10. The Overall Frequency of Spontaneous Puberty

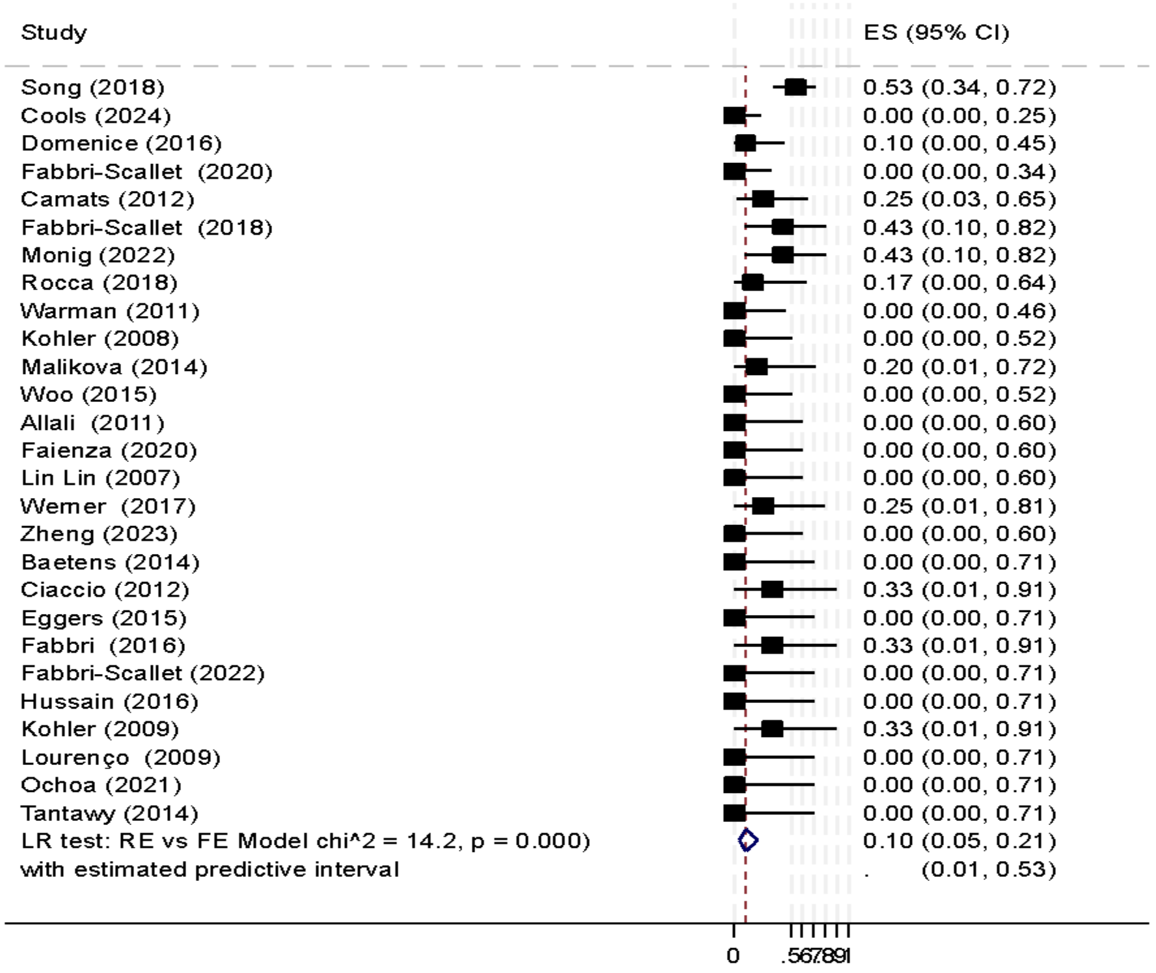

Figure 11. The Overall Frequency of Gender Change
